# Association of systemic immune-inflammation index and aggregate index of systemic inflammation with clinical status in stable and exacerbated COPD: A single-center retrospective study

**DOI:** 10.1097/MD.0000000000044589

**Published:** 2025-09-26

**Authors:** Ayshan Mammadova, Nurgul Naurzvai

**Affiliations:** a Department of Chest Diseases, Faculty of Medicine, Lokman Hekim University Akay Hospital, Ankara, Turkey; b Department of Chest Diseases, Faculty of Medicine, Acibadem University, Istanbul, Turkey.

**Keywords:** aggregate index of systemic inflammation (AISI), chronic obstructive pulmonary disease (COPD), exacerbation, inflammation, systemic immune-inflammation index (SII)

## Abstract

Chronic obstructive pulmonary disease (COPD) is a condition characterized by persistent airflow limitation and inflammation. Exacerbations may occur even during the stable phase of COPD, which significantly affects patients’ quality of life. This study investigates the association of the Systemic Immune-Inflammation Index (SII) and the Aggregate Index of Systemic Inflammation (AISI) with clinical status in COPD patients during stable and exacerbation phases. This retrospective study included 245 patients diagnosed with COPD between 2020 and 2025. The patients were divided into 2 groups: stable COPD (n = 120) and COPD exacerbation (n = 125). The demographic characteristics, pulmonary function tests, and biomarkers (SII, AISI) of the patients were retrospectively analyzed. Statistical significance level was accepted as *P* < .05. In the exacerbation group, the SII and AISI values were significantly higher compared to the stable group (SII: 2782.32 ± 439.56, AISI: 1242.6 ± 239.91, *P* < .001). A strong positive correlation was found between SII and AISI with hospital admission in the exacerbation group (SII: *R* = 0.824, AISI: *R* = 0.956, *P* < .001). In the ROC analysis performed to predict the exacerbation group, cutoff values of 688.8 for SII and 397.56 for AISI yielded AUC values of 0.962 and 0.938, respectively. Multivariate logistic regression analysis revealed that both SII (OR: 1.001, 95% CI: 1.000–1.002, *P* = .031) and AISI (OR: 1.003, 95% CI: 1.001–1.005, *P* = .002) serve as independent predictors of COPD exacerbation. SII and AISI are useful biomarkers associated with the clinical status of COPD patients in both stable and exacerbation phases. High SII and AISI values can accurately predict exacerbation and hospital admission risk, thus aiding in the more effective management of treatment processes.

## 1. Introduction

Chronic obstructive pulmonary disease (COPD) is a progressive respiratory condition characterized by persistent airflow limitation and chronic airway inflammation, significantly affecting patients’ quality of life and increasing global mortality rates.^[1]^ Exacerbations can occur even during the stable phase of COPD, worsening clinical status, increasing hospital admissions, and accelerating disease progression.^[2,3]^ Therefore, predicting exacerbation risk during the stable phase is crucial for optimizing treatment and improving patient outcomes.

In recent years, several biomarkers and clinical parameters related to inflammation and immune response have been investigated for their potential to predict exacerbation risk in stable COPD patients. The Systemic Immune-Inflammation Index (SII), initially introduced as a prognostic tool in oncology, reflects systemic inflammation and has been associated with disease prognosis in various conditions, including hepatocellular carcinoma and lupus nephritis.^[4,5]^

Similarly, the Aggregate Index of Systemic Inflammation (AISI) is a novel biomarker calculated using neutrophils, monocytes, platelets, and lymphocytes, which has demonstrated prognostic relevance in diseases such as stroke, hypertension, and idiopathic pulmonary fibrosis.^[6,7]^

This study aims to investigate the association of SII and AISI values with the clinical status of patients with stable COPD and those admitted due to exacerbation.

## 2. Materials and methods

**Figure 1.**
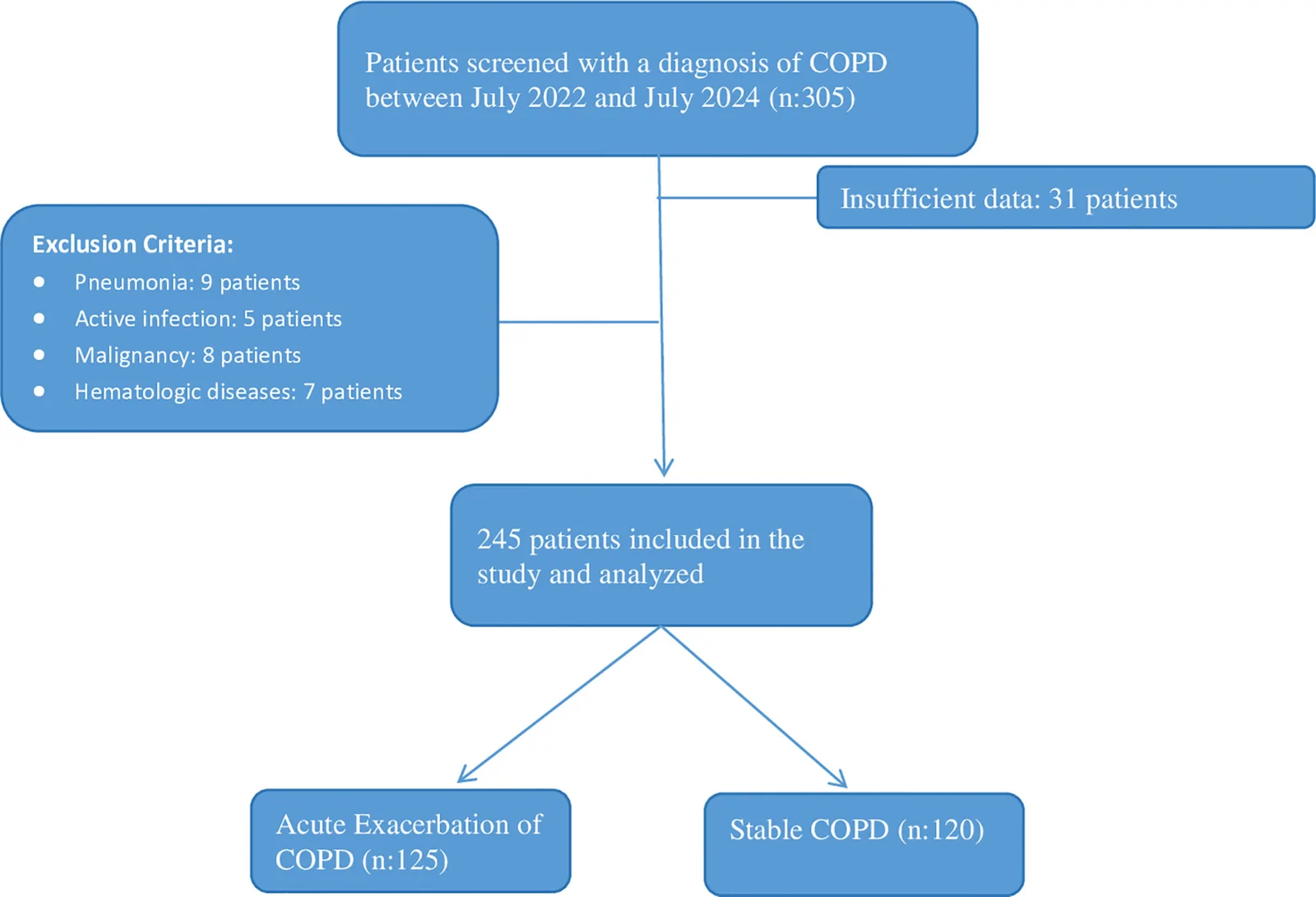
Flowchart of patient selection process in the study. COPD = chronic obstructive pulmonary disease.

This single-center retrospective study included a total of 245 patients diagnosed with COPD who presented to our hospital between July 1, 2022, and July 1, 2024 (Fig. 1). The study was approved by the Clinical Research Ethics Committee of Lokman University Faculty of Medicine on April 28, 2025, with approval number 103. The diagnosis of COPD was confirmed with post-bronchodilator tests, in addition to the clinical symptoms and smoking history of the patients. During the diagnostic process, patients with a post-bronchodilator FEV1/FVC ratio below 0.7 were classified as having COPD.^[8]^

The patients were divided into 2 main groups: stable COPD and COPD exacerbation. COPD exacerbation diagnosis was made based on the symptoms defined in the current Global Initiative for Chronic Obstructive Lung Disease (GOLD) report.^[8]^ Patients were classified into 2 main groups: stable COPD and COPD exacerbation. COPD exacerbation was defined according to the current Global Initiative for Chronic Obstructive Lung Disease (GOLD) criteria as an acute worsening of respiratory symptoms – such as increased dyspnea, sputum volume, or sputum purulence – that requires additional treatment (e.g., antibiotics or corticosteroids) and/or hospitalization.^[8]^ The stable COPD group consisted of patients attending outpatient clinics or pulmonary rehabilitation units for routine follow-up or treatment, with no signs of acute exacerbation at presentation. These patients had not experienced exacerbation episodes or received systemic corticosteroids or antibiotics within the preceding 4 weeks, ensuring clinical stability.

The patients’ demographic characteristics (age, gender), smoking history, comorbidities, symptoms, pulmonary function tests (Forced Expiratory Volume in 1 second (FEV1%), Forced Vital Capacity (FVC%), FEV1/FVC), oxygen saturation in room air at presentation, exacerbations, and hospitalizations in the past year, mMRC scores, GOLD stages, and some laboratory parameters at the time of admission (leukocyte, monocyte, eosinophil, lymphocyte, neutrophil, C-Reactive Protein (CRP), albumin, SII, AISI), and the use of respiratory support devices (oxygen concentrators, noninvasive mechanical ventilation) were retrospectively collected from the hospital’s information system, patient files, and archived records. The study included patients aged 18 years and above who were diagnosed with COPD and had complete blood parameters at the time of admission. Patients with respiratory diseases other than COPD, missing data, clinically and radiologically confirmed pneumonia, active infections, a history of malignancy, or hematologic disorders were excluded from the study (Fig. 1).

Complete blood count was analyzed in our hospital laboratory using the spectrophotometric/impedance method (Beckman Coulter LH 780 Analyzer; Beckman Coulter, Inc.). The SII value was calculated using the formula (absolute platelet count × absolute neutrophil count)/ absolute lymphocyte count. AISI was calculated using the formula [(neutrophil × platelet × monocyte/ lymphocyte)].

### 2.1. Statistical analysis

Statistical analyses were performed using IBM SPSS version 26.0. Continuous variables were expressed as mean ± standard deviation or median (min–max), and categorical variables as frequencies and percentages. Normality was assessed using the Kolmogorov-Smirnov and Shapiro–Wilk tests. Depending on data distribution, comparisons between 2 groups were performed using the independent t-test or Mann–Whitney U test, and comparisons among multiple groups were conducted using one-way ANOVA with Bonferroni post-hoc analysis. Categorical variables were compared using the Chi-square test. Pearson correlation was used to evaluate associations. ROC analysis was performed to identify optimal cutoff values of SII and AISI for predicting exacerbations. Univariate and multivariate logistic regression analyses were used to identify independent predictors. Model fit was assessed with the Hosmer-Lemeshow test, and explanatory power was evaluated using Nagelkerke R². Statistical significance level was accepted as *P* < .05.

A post-hoc power analysis was performed using the sample sizes of 125 (exacerbation group) and 120 (stable COPD group). Based on the observed effect sizes (Cohen d = 5.67 for SII and 1.41 for AISI), the study has a statistical power >99% to detect differences between groups at an alpha level of 0.05.

## 3. Results

A total of 245 patients were included in our study, with 120 in the stable COPD group and 125 in the COPD exacerbation group. The overall mean age of all patients was 66.6 ± 8.9, with the mean age of the exacerbation group being 67.6 ± 9.4 and that of the stable COPD group being 65.5 ± 9.1. Of all patients, 198 (80.8%) were male, and 37 (19.2%) were female. No statistically significant difference was observed between the 2 groups in terms of mean age (*P* = .211) (Table 1), but a significant difference in gender distribution was found (*P* = .045), with a higher proportion of male patients in the exacerbation group. Regarding smoking history, 91.2% of the exacerbation group and 88.7% of the stable disease group had a history of smoking, and no statistically significant difference was found between the 2 groups in terms of smoking history (*P* = .346). When the mMRC scores of patients were examined, it was found that the exacerbation group had higher scores compared to the stable group (*P* < .001). The SII and AISI values of the patients included in the study were found to be 1914.6 ± 255.86 and 1055.26 ± 150.56, respectively. SII and AISI values were significantly higher in the exacerbation group compared to the stable COPD group (*P* < .001) (Table 1).

**Table 1:** Clinical characteristics and laboratory parameters of exacerbation and stable COPD groups.

	Exacerbation group n = 125	Stable COPD group n = 120	*P*-value
Age	67.6 ± 9.4	65.5 ± 9.1	.211*
Sex (male, n%)	110 (%88)	88 (%73)	.045†
mMRC	2.88 ± 1.17	1.72 ± 0.68	.001
Number of hospital admissions in the last year	2.23 ± 1.33	0.36 ± 0.69	.001
Number of exacerbations in the last year	2.46 ± 1.15	0.44 ± 0.79	.001
FEV1%	43.07 ± 19.21	63.07 ± 11.34	.001
FVC %	61.23 ± 19.12	67.54 ± 21.12	.054
FEV1/FVC	51.42 ± 6.12	63.44 ± 6.12	.035
Neutrophil (x10^9^/L)	8.97 ± 3.21	4.26 ± 1.32	.001
Lymphocyte (x10^9^/L)	1.37 ± 0.42	2.42 ± 0.66	.001
Monocyte (x10^9^/L)	0.56 ± 0.18	0.54 ± 0.12	.402
PLT (x10^9^/L)	376.77 ± 89.23	264.35 ± 55.77	.003
CRP (mg/L)	32.24 (1.57–215)	5.13 (0.29–78.4)	.001‡
SII	2782.32 ± 439.56	836.62 ± 197.77	.001
AISI	1531.24 ± 1007.49	499.78 ± 204.49	.001

AISI = the aggregate index of systemic inflammation, COPD = chronic obstructive pulmonary disease, CRP = C-reactive protein, FEV1 = forced expiratory volume in 1 second, FVC = forced vital capacity, mMRC = modified medical research council, PLT = platelet count, SII = systemic immune-inflammation index.

* t-testi.

† ki-kare analizi.

‡ Mann–Whitney *U* testi.

Among the patients admitted due to COPD exacerbation, 117 (47.8%) were hospitalized, 110 (44.9%) were treated in the ward, and 7 (2.9%) were monitored in the intensive care unit. The SII and AISI values of patients monitored in the intensive care unit (2964.9 ± 1905.05; 1610.9 ± 9) were found to be significantly higher than those of patients treated in the ward (1380.4 ± 829.9; 784.5 ± 583.2) (*P* < .001, *P* < .001).

The correlations between hospitalization status and the SII and AISI values were evaluated using Pearson correlation analysis. A positive correlation was found between hospitalization status and both SII (*P* < .001, *R* = 0.824) and AISI (*P* < .001, *R* = 0.956). In the COPD exacerbation group, significant positive correlations were observed between serum SII and AISI (*P* < .001, *R* = 0.775), neutrophils (*P* < .001, *R* = 0.312), CRP (*P* = .002, *R* = 0.423), mMRC score (*P* = .002, *R* = 0.492), and CAT score (*P* = .006, *R* = 0.429). Negative correlations were also identified between SII and AISI with FEV1 (*P* = .003, r = -0.466), SPO2 (*P* = .003, r = -0.234), and albumin (*P* = .004, r = -0.368).

When the patients were classified according to the GOLD staging system, 16.7% (n = 41) were classified as GOLD A, 31.4% (n = 77) as GOLD B, and 51.8% (n = 127) as GOLD E. Significant differences were found in neutrophil counts among the groups (*P* = .003), driven by higher values in GOLD E compared to GOLD A and B. Similarly, SII and AISI values were significantly elevated in GOLD E patients (Table 2).

**Table 2:** Comparison of clinical parameters and SII and AISI values according to GOLD stages in COPD patients.

	A	B	E	*P*-value*
BMI	27.65 ± 5.67	25.77 ± 3.44	26.66 ± 4.56	.567
FEV1 %	61.45 ± 17.66^a^	58.75 ± 21.33^b^	42.27 ± 13.79^a,b^	.003
FVC %	76.23 ± 20.67^a^	71.24 ± 22.45^b^	56.78 ± 17.45^a,b^	.015
FEV1/FVC	63.34 ± 7.14	59.17 ± 8.3	53.44 ± 9.72	.032
Neutrophil (x10^9^/L)	8.29 ± 3.23^a^	7.19 ± 3.25^b^	10.16 ± 2.22^a,b^	.003
Lymphocyte (x10^9^/L)	2.16 ± 0.81^a^	2.07 ± 0.77^b^	1.24 ± 0.64^a,b^	.047
Monocyte (x10^9^/L)	0.28 ± 0.03	0.35 ± 0.04	0.37 ± 0.11	.788
PLT (x10^9^/L)	277.37 ± 79.23	271.37 ± 86.56	281.44 ± 77.27	.745
SII	894.16 ± 639.62^a^	1040.13 ± 899.9^b^	3518.43 ± 1783.92^a,b^	.001
AISI	546.07 ± 467.6^a^	664.6 ± 711.75^b^	1806.95 ± 1108.24^a,b^	.002

AISI = the aggregate index of systemic inflammation, BMI = body mass index, COPD = chronic obstructive pulmonary disease, FEV 1 = forced expiratory volume in 1 second, FVC = forced vital capacity, GOLD = global initiative for chronic obstructive lung disease, PLT = platelet count, SII = systemic immune-inflammation index.

* ANOVA Test, ^a,b^Bonferroni post-hoc test.

**Figure 2.**
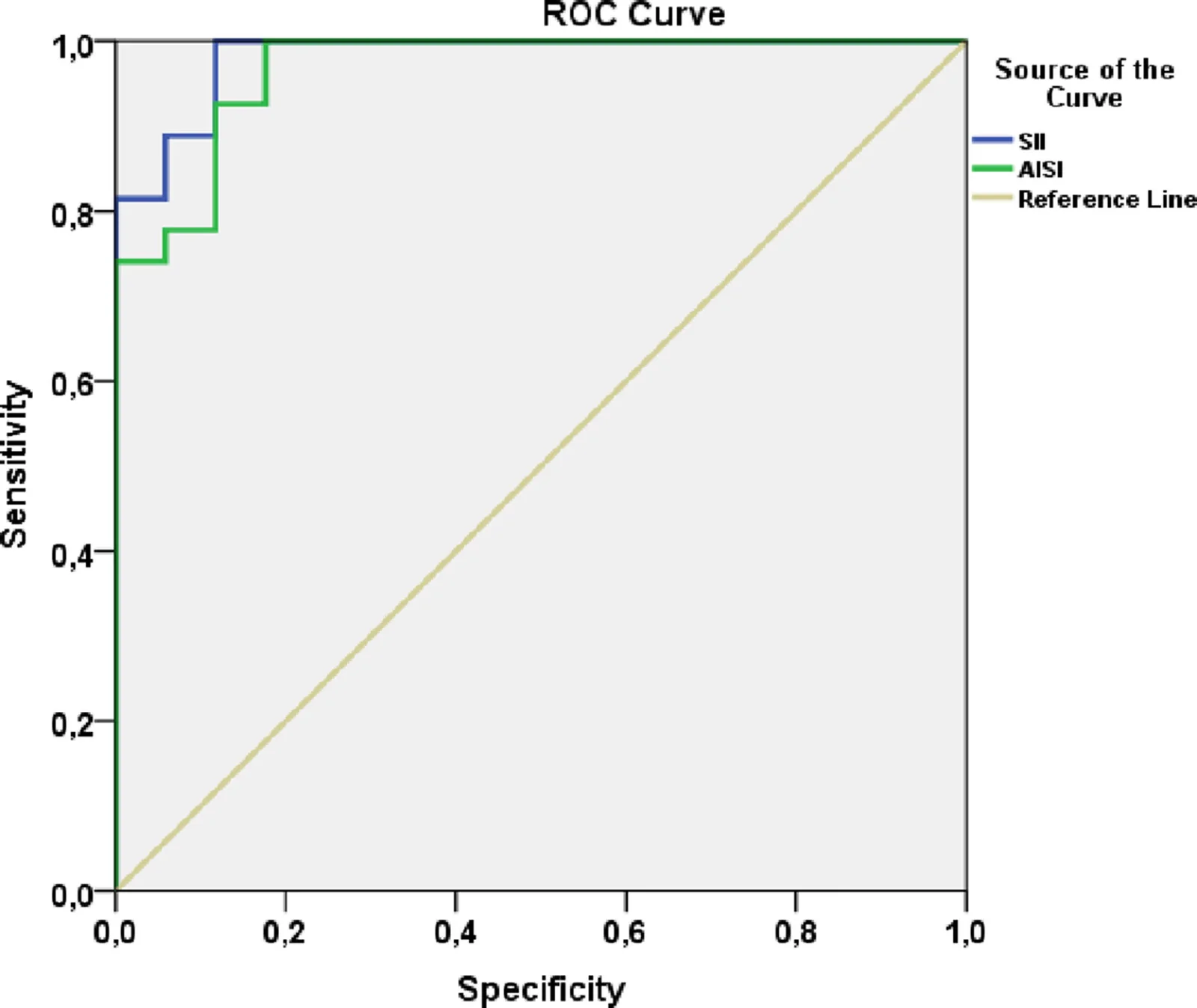
ROC analysis of SII and AISI indices between stable and exacerbation groups in COPD patients. The analysis includes 120 patients in the stable COPD group and 125 patients in the exacerbation group. AISI = aggregate index of systemic inflammation, COPD = chronic obstructive pulmonary disease, ROC = receiver operating characteristic, SII = systemic immune-inflammation index.

Significant cutoff values that could predict the exacerbation group were analyzed. Accordingly, when the cutoff for SII was 688.8, 100% sensitivity and 88.2% specificity were obtained (Youden index = 0.882). When the cutoff for AISI was 397.56, 96.3% sensitivity and 82.6% specificity were obtained (Youden index = 0.789) (Table 3, Fig. 2). In COPD, elevated SII and AISI were found to be predictive of exacerbation and severe disease.

**Table 3:** ROC analyses between stable and exacerbation states in COPD: diagnostic performance of SII and AISI.

	AUC (%95 CI)	*P*-value	Cutoff	Sensitivity	Specificity
SII	0.983 (0.953–1.000)	.001	688.8	100	88.2
AISI	0.967 (0.922–1.000)	.001	397.56	96.3	82.6

AISI = the aggregate index of systemic inflammation, AUC = area under the curve, CI = confidence interval, COPD = chronic obstructive pulmonary disease, ROC = receiver operating characteristic, SII = systemic immune-inflammation index.

To assess the relationship between clinical and inflammatory variables that could predict COPD exacerbation, univariate logistic regression analysis was performed (Table 4). This analysis identified that certain variables, including age, SII, AISI, CRP, FEV₁, and mMRC scores, were statistically significantly associated with COPD exacerbation. These significant variables from the univariate analysis were then included in the multivariate logistic regression analysis to evaluate their independent relationship with COPD exacerbation. In the multivariate logistic regression analysis, 2 separate models were constructed. In Model 1, SII was identified as an independent predictor of COPD exacerbation (OR: 1.001, 95% CI: 1.000–1.002, *P* = .031). Similarly, in Model 2, AISI was found to be an independent predictor associated with COPD exacerbation (OR: 1.003, 95% CI: 1.001–1.005, *P* = .002). These findings suggest that elevated SII and AISI values may be associated with increased disease severity in COPD and could potentially serve as supportive markers for clinical assessment.

**Table 4:** Logistic regression analysis of predictors of COPD exacerbation.

Univariate logistic regression analysis results		
Variable	OR (95% CI)	*P*-value
Age	1.155 (1.04–1.29)	.034
SII	1.122 (1.101–1.203)	.001
AISI	1.224 (1.102–1.298)	.001
Sex (male)	1.757 (1.017–3.049)	.055
CRP	1.085 (1.031–1.143)	.003
FEV1, %	0.949 (0.911–0.983)	.004
SpO2	0.912 (0.866–0.978)	.122
Albumin	0.82 (0.713–0.947)	.236
mMRC	1.637 (1.291–2.064)	.001
Corticosteroid use	1.104 (0.802–1.467)	.421
Eosinophil count	1.012 (0.975–1.050)	.312

Multivariate logistic regression analysis – model 1		
Age	0.945 (0.881–0.999)	.344
SII	1.001 (1.000–1.002)	.005
CRP	1.04 (1.00–1.09)	.178
FEV1, %	0.96 (0.92–0.99)	.012
mMRC	1.41 (1.10–1.81)	.006

Multivariate logistic regression analysis – model 2		
Age	0.934 (0.873–0.997)	.357
AISI	1.003 (1.001–1.005)	.002
CRP	1.032 (0.994–1.088)	.067
FEV1, %	0.955 (0.915–0.997)	.010
mMRC	1.351 (1.051–1.74)7	.018

AISI = aggregate index of systemic inflammation, CI = confidence interval, COPD = chronic obstructive pulmonary disease, CRP = C-reactive protein, FEV₁,% = forced expiratory volume in 1 second (percent predicted), mMRC = modified medical research council dyspnea scale, OR = odds ratio, SII = systemic immune-inflammation index, SpO₂ = peripheral oxygen saturation.

## 4. Discussion

COPD is a disease characterized by inflammation both in its stable phase and during exacerbations. Inflammation initially begins in the airways through inflammatory cells and various mediators involved in inflammation. This study demonstrated that systemic inflammation indices, specifically the SII and the AISI, are significantly elevated in patients experiencing COPD exacerbations compared to those with stable disease. These markers showed strong correlations with clinical severity, hospitalization rates, and established prognostic indicators, highlighting their potential as valuable tools for early identification and management of exacerbations in COPD.

Studies have shown varying results regarding the gender distribution of COPD. In a study conducted by Özkaya et al., the male proportion was found to be 80.2%,^[9]^ while in a large-scale study by Vogelmeier et al., the male proportion was reported to be 54%.^[10]^ Both in the literature and in our own study, it is evident that COPD is more common in men.

In a retrospective study by Kalemci et al., a significant increase in platelet distribution width and platelet-to-lymphocyte ratio values was observed as the severity of COPD increased^[11]^; in another study, a negative correlation was found between neutrophil-to-lymphocyte ratio and FEV1, while a positive correlation was noted with the dyspnea score.^[12]^ Similar results were obtained in our study.

In a study involving 10,037 individuals diagnosed with COVID-19, it was reported that the SII values of hospitalized patients were significantly higher compared to those receiving outpatient treatment. The same study also mentioned that patients who died from COVID-19 had higher SII values compared to those discharged.^[13]^ Another study showed that higher SII levels were associated with COPD prevalence and increased mortality risk from all causes in COPD patients.^[14]^ Since our study was a retrospective survey, we did not have access to mortality data for all patients and therefore could not assess the role of SII and AISI in predicting mortality. However, it was observed that these indices were higher in the COPD exacerbation group and in hospitalized patients compared to those monitored on an outpatient basis. It has been concluded that SII has potential as a prognostic tool in the management of acute respiratory distress syndrome patients, and incorporating this parameter into clinical practice may assist in individualizing treatment strategies.^[15]^

AISI, a composite index based on neutrophils, monocytes, platelets, and lymphocytes, has been reported to perform strongly in predicting the severity of inflammation and disease prognosis in many studies.^[16,17]^ In a study involving patients with Idiopathic Pulmonary Fibrosis, AISI was shown to better reflect the inflammatory state and could allow for the early identification of patients at risk of death.^[6]^ Additionally, it was emphasized that AISI could be a superior index compared to simpler markers as a predictive tool. In another study, it was noted that the novel hematological markers, SII and AISI, played an important role in evaluating inflammation and detecting high disease activity in patients with systemic lupus erythematosus. In this study, significant positive correlations were observed between disease activity and SII and AISI, with values of (*R* = 0.265, *P* = .011) for SII and (*R* = 0.309, *P* = .003) for AISI, respectively.^[18]^ Similarly, a retrospective study on influenza B patients reported significantly higher SII and AISI levels in hospitalized individuals compared to outpatients, with both indices showing strong positive correlations with hospitalization.^[19]^

It has been shown that AISI can be used to indicate the severity of disease and the need for intensive care in Coronavirus disease 2019 (COVID-19) infections,^[20]^ and also serves as a significant marker in detecting aortic valve sclerosis in patients with hypertension.^[21]^ In COVID-19 patients with COPD, AISI was found to play a reliable role in predicting mortality (HR = 2.010, 95% CI = 1.048–3.855, *P* < .05).^[22]^

## 5. Conclusion

This study demonstrates that the SII and AISI indices are significantly associated with inflammatory status and disease severity in stable and exacerbated COPD patients. The finding that these indices were significantly higher in the exacerbation group and that higher values were observed in hospitalized patients suggests that incorporating these parameters into clinical practice may help individualize treatment strategies. Additionally, it was concluded that these biomarkers could accurately predict exacerbations and hospitalization rates in COPD. Future studies should examine the effects of SII and AISI in larger populations and on long-term outcomes such as mortality.

### 5.1. Limitations

This study has several important limitations. Firstly, as a retrospective cohort study, it was not possible to obtain long-term mortality data and clinical follow-up for some patients, preventing the assessment of the effects of SII and AISI on mortality. Additionally, being a single-center study limits the generalizability of the findings to the broader population. Furthermore, the cutoff values for SII and AISI were determined based on ROC analysis within our study cohort using the Youden index, but these thresholds were not internally or externally validated. This may increase the risk of overfitting, and thus these results should be interpreted with caution. For these reasons, the findings need to be confirmed in larger, multicenter, and prospective studies.

## Author contributions

**Conceptualization:** Ayshan Mammadova, Nurgul Naurzvai.

**Data curation:** Ayshan Mammadova, Nurgul Naurzvai.

**Formal analysis:** Ayshan Mammadova.

**Investigation:** Ayshan Mammadova.

**Methodology:** Ayshan Mammadova, Nurgul Naurzvai.

**Writing – original draft:** Ayshan Mammadova, Nurgul Naurzvai.

**Writing – review & editing:** Ayshan Mammadova.
